# Evaluation Protocol for Analogue Intelligent Medical Radars: Towards a Systematic Approach Based on Theory and a State of the Art

**DOI:** 10.3390/s23063036

**Published:** 2023-03-11

**Authors:** Geoffray Battiston, Rémi Régnier, Olivier Galibert

**Affiliations:** Laboratoire National de Métrologie et D’essais (LNE), 78197 Trappes, France

**Keywords:** vital parameters, machine learning, radar theory, analogue AI, evaluation protocol

## Abstract

We propose the basis for a systematised approach to the performance evaluation of analogue intelligent medical radars. In the first part, we review the literature on the evaluation of medical radars and compare the provided experimental elements with models from radar theory in order to identify the key physical parameters that will be useful to develop a comprehensive protocol. In the second part, we present our experimental equipment, protocol and metrics to carry out such an evaluation.

## 1. Introduction

The present work takes place within the framework of the AIR CHIST-ERA project [1,2]. Its aim is to prototype and evaluate the performance of an analogue intelligent chip for short and middle range medical radar signal processing. 

Medical radars can measure the human heart and breathing rates and have the advantage of working without contact with the skin, at a distance of up to several meters. They have already been the topic of many publications (see, for instance, the review articles [3,4,5] or the book [6] for a general coverage from a medical viewpoint), and we will analyse some of them in the literature review in this paper. Most publication authors present their radars and detail their mathematical algorithms to derive the breathing and heart rates. They also construct experiments with their hardware as a proof of concept, and we point out that many of them have their own protocol. Sometimes, they perform a statistical study to measure the difference between the results of their algorithm and the baseline measurements performed on the human body [7,8].

The aim of this paper is to advance the topic of medical radar evaluation, more particularly for analogue intelligent radars. Because of the variety of evaluation protocols found in the literature, we wanted, in the first part of this paper, to summarise all of the interesting physical quantities involved in the literature on medical radar evaluation, and compare them with those of radar theory models, in order to take into account as many physical elements as possible to systematise the evaluation and develop a comprehensive protocol within a physical–mathematical framework.

Intelligent radars integrate artificial intelligence, i.e., a signal-processing algorithm whose parameters have been derived with the help of a database and some statistical optimization algorithms. Such an approach provides the digital signal processor the ability to potentially change its parameters within a given signal analysis structure. Analogue intelligent radars go further by replacing the digital signal processor with an analogue integrated circuit: instead of performing calculations through a complex computer structure in the digital domain, the whole processing process happens in the analogue domain in one or multiple very-low-power ASICs. This technology aims at increasing the radar signal processing speed and decreasing the energy consumption (multiple applications for AI can be found in [9,10,11,12,13,14,15,16,17]). The protocol should therefore also control these elements.

In addition to the development of a protocol, and in order to be sure that the results given by an analogue intelligent medical radar are correct, we need to acquire reference measurements and to develop metrics to compare the results. We present such elements in the second part of this paper. We warn in advance that we do not yet have the analogue intelligent radar prototype from our research project, so we have not been able to provide all the details required for the metrics.

Overall, this work aims to be a first step towards a more comprehensive and systematic approach to the evaluation of analogue intelligent medical radars, with a protocol built from both theory and practice and metrics adapted to analogue intelligent radars.

We present our work as follows. Section 2 introduces the concept of intelligent radars and the principle of their evaluation. Section 2.1. offers a state of the art about the evaluation of medical radars. Section 2.2. is a very short introduction to radar theory. Section 2.3. makes the link between the previous two sections in order to identify important physical quantities to monitor during the evaluation of the radar. 

After these four theoretical sections, we are able to propose, for a second time, an experimental setup, a protocol and metrics to achieve the evaluation of an analogue intelligent medical radar. In Section 3, we present the hardware we used to assemble a prototype to measure reference values of heart and breathing rates. Section 4.1 details the protocol with multiple possible scenarios. Section 4.2. addresses the metrics issue. Finally, in Section 5, we discuss possible directions to investigate in order to improve our current work, which is not complete. 

## 2. Intelligent Radars: What Do We Evaluate and How? ”I Confirm”

For the sake of simplicity, in the introduction, we use the term radar in a broad sense. In practice, it contains two main elements: (1) the transmission/reception antennas and attached circuitry and (2) the calculus entity processing the signals measured from the first part.

System (1) is defined by its power supply (consumption W), its wave emission/reception (antenna diagram, number of reception antennas, kind of emission (UWB, CW, FMCW), frequencies, power) and its signal processing (time of calculation, resolution). Depending on these elements, we can find multiple strategies linked to system (2) in the literature to extract interesting data, including heart and breathing rates (see, for instance, [18,19,20,21]).

Compared to these classical radar technologies, intelligent radar integrates a computation entity that performs an AI calculation on the result of the received signal. In project AIR, this entity is an analogue intelligence circuit that computes the heartbeat and respiratory frequencies from the information that system (1) sends. Therefore, there are three levels of information:

1. The measurements directly performed on a target.
2. A signal information sent by the radar acquisition circuit.
3. The results given by the AI after the processing of this information.

A thorough evaluation would aim at evaluating the quality of the correspondence between levels (1) and (2), (2) and (3), (1) and (3). In the scope of our research, we only focus on the last two correspondences because the first one concerns the antenna hardware, which we do not evaluate. Moreover, in the scope of the present paper, we will only focus on the (1)–(3) relationship, between what we measure on a target and what the analogue intelligent radar gives as a result. In order to do this, we need to define what a target is and in which physical context it operates. This is what Section 2.1 and Section 2.2 propose.

### 2.1. State of the Art

In this section, we analyse more than 50 papers on medical radar experiments in order to extract from them interesting elements about their protocols [22,23,24,25,26,27,28,29,30,31,32,33,34,35,36,37,38,39,40,41,42,43,44,45,46,47,48,49,50,51,52,53,54,55,56,57,58,59,60,61,62,63,64,65,66,67,68,69,70,71,72,73,74,75,76,77].

In almost of all these papers, we can find information on the radar and the target positioning: the distance between them is given, and the target may have various positions and movements. For instance, [22,23,34,40] show a sitting target, [28] show a static standing target, [24,25,71] display a lying-down target, and [45,61] present a walking target. This information belongs to the ‘Positioning’ category.

Besides the positioning, we can find data on the nature and number of targets. In most of these papers, the target is singular, but there is sometimes mention of multiple individual targets being tested [34,41,48,53,64,69,76,77], or two targets at the same time [42,46,49,52,61], or even more [33]. Sometimes, the authors present data about the targets’ age and weight [8,53] or torso dimensions [41], or gender [76]. The target can also be a machine simulating human respiration [32,47,50,51,72,73]. In a few other papers [38,45,47], it is specified that the targets wear clothes. We classify these elements in the category ‘Target’ (see Table 1).

We can also find details on the experimental environment, including the presence of walls between the radar and the target [43,44] or around them [32,48,61]. These walls can also be anechoic [39,45,66]. Rarely, the experiment is carried out outside with no proximate walls [35,36,37,38,39,40,41,42,43,44,45,46,47,48,49,50,51,52,53,54,55,56,57,58,59], or surrounded by ruins [24,74]. Ref. [26] shows that there can be absorption in the atmosphere, and [53] shows that there can be speaking noise. All of these elements enter the ‘Environment’ category.

For the last protocol information category, we deal with the ‘Measurements’ themselves. Details can be given about the sensors for measuring heart and respiration rates [7,8,27,30,40,48,57,65,76], the duration of the experiment [18,19,42,52,55,65,69,73], and the breathing style [22,36,48,56,60,65,75]. The Bland–Altman method to compare sensors and radar data is sometimes applied [34,76].

These four categories are visible in Table 1. We wanted to provide an in-depth analysis so that interested readers could visibly observe particular experimental configurations. When not specified, the target is human, is facing the radar, the radar aims at the target’s thoracic cage and is at the same altitude, the distance is between the target and the radar antenna, and the atmosphere is a laboratory one. For experiments carried out on multiple persons, but not at the same time, we will use the term “individual”. In many of these papers, the type of radar transmission and frequency are given, but we do not reproduce them here since our goal is not to focus on these elements.

**Table 1:** Literature review. Articles are cited in the first column.

Article	Target	Positioning	Environment	Measurements
[7]	14 individual targets (age and weight given) or multiple targets.	8 m. Different movements are studied.	Wall	AlicePDX sensors. 120 s duration.
[8]	Age, weight	Sitting at 1.5 m		Spirometer
[18]		1 m	Wall	35 s duration
[19]		30 cm		30 s duration
[20]			Wooden wall	
[22]		Sitting at 0.3 m		Shallow breathing
[23]		Sitting at 1.5 m		
[24]		Lying down on the ground	Building. Three 20 cm floors between.	
[25]		Lying down on back or belly. Radar on the room ceiling.		
[26]	Body absorption		Atmosphere absorption	
[27]				Various sensors are presented
[28]		Static standing target at 1 m		
[29]		10 cm		
[30]		Radar on ceiling at 2.5 m height. Lying down target below it.		ECG device
[31]	Multiple targets			
[32]	Translating plate		$4.5\;\times \;5\;m^{2}$ room	
[33]	4	Less than 2.5 m and lying down		Oximeters
[34]	17 individual	Sitting on a bed		30 s duration with a spirometer. Bland–Altman to compare radar and reference measurements.
[35]	One with a blanket	Lying down. Radar is tied to a moving or static UAV at 2 m altitude.	Outside	
[36]		Radar 20 cm above lying down target. The latter has various positions		Breathing driven with metronome
[37]		30 cm to 69 m		
[38]	Clothes	2 m distance		
[39]			Anechoic walls	
[40]		Sitting at 1 m		Chest belt
[41]	22 individual (torso dimensions)	0.5–2 m		Three-lead electrocardiogram. Piezoelectric respiratory effort belt.
[42]	2	Sitting at 1.5 m		2 min duration. Neulog NUL236 respiration belt and a Neulog NUL208 heartrate sensor
[43]		1 m	8 cm wall between	
[44]		10 m	Wall between	
[45]	Clothes	Walking. Radar with various angles.	Anechoic chamber	
[46]	2	Same distance for both targets, but different angular position.		
[47]	Various clothes. Puppet.	3–4 m distance. Standing with various body angles.		
[48]	10 individual		205 × 120 × 195 m^3^ room	Wireless respiration monitor and ECG. Normal and hold respiration.
[49]	2	TR couple radar at 2 m. Sitting or lying down.		
[50]	Oscillating plate			
[51]	Thorax emulator	1 m		
[52]	2	Lying down in various positions. Radar 1 m above.	Speaking	3 min duration with ECG + belt sensors
[53]	8 individual (age and weight given)	Radar fixed on back of a wheeling chair.		
[54]		Target sitting behind a 10 cm wall		ECG
[55]		Sitting at 0.75 m		ECG. 3 min duration.
[56]				ECG. Normal and hold respiration.
[57]		Sitting at 1.5 m		Spirometer
[58]	Baby	Lying down with radar 35 cm above		
[59]		Walking up to 37 m	Tennis court	
[60]		Sitting at 2.5 m	Wall between	Hold respiration
[61]	2	Walking	Wall behind at 19 m	
[62]			Wall	
[63]		5 to 15 m		
[64]	10 individual	Sitting of lying down with various body angles. Radar on ceiling.		
[65]		Sitting target from 1 m to 50 m		Various respirations. 2 min. BioRadio150 sensor.
[66]	Animal	20 cm	Anechoic wall	
[67]	Adult	1 m		
[68]	Multiple	Sitting at 1 m		
[69]	50 individual	Sitting at 1 m		3 min duration
[70]		Lying down at 1 m		
[71]		Lying down. Radar above.		Biopac sensor
[72]	Metal plate	1 m	Wooden wall between	
[73]	Breathing simulation machine and human	Sitting at various distances around the meter		1 min duration
[74]			Building and ruins	
[75]		Sitting at 1 m	3 m × 3 m room	Various respirations. (normal, deep quick, deep, quick, hold)
[76]	10 individual (age, gender)	Different lying positions		Embla Titanium sensor. 2 min. Before and after sport. 40 s FFT window for signal analysis. Bland–Altman comparison.
[77]	30 individuals (age, body, mass)	Supine position. Radar 1 m above.		Task Force Monitor from CNSystems

This literature review provides information about various categories of details for describing a measurement experiment on a medical radar:

Target detail:

- Number of targets;
- Nature of the target;
- Age, height, weight of the target, if human;
- Target clothes.

Positioning detail:

- Distance and position of the target;
- Movement of the target;
- Alignment of the radar;
- Position of the radar.

Environment details:

- Presence of an obstacle;
- Atmosphere quality;
- Speaking;
- Room (of given dimension) or exterior;
- Walls.

Measurements details:

- Duration of an experiment;
- Body sensors for comparison;
- Kind of respiration;
- Reference sensors;
- Metrics to compare radar data with body sensors.

In these papers, the comparison between radar measurements and reference measurements (mainly using a respiratory belt and ECG), when conducted, is mainly performed in the frequency domain by comparing the location of the main frequency of the Fourier transform of the two signals.

Ref. [64] shows an interesting result: the maximal distances for measuring heart and breathing rates may not be the same (probably because of the amplitude difference).

The authors believe that the diversity of protocols motivates the creation of a more systematic and comprehensive medical radar evaluation architecture. In the next section, we want to make sure that we have not missed anything useful for the evaluation by using radar theory. 

### 2.2. Radar Theory

The following section aims to briefly recall the important results of radar physics, as explained in radar theory books [78,79,80,81], and highlights the physical quantities involved.

#### 2.2.1. Radar Detection in the Perfect Case

A transmitter antenna emits an electromagnetic radar wave from an electric circuit signal. The receiving antenna converts the reflected electromagnetic signal into an electric circuit signal. In practice, there are differences in phase, frequency, amplitude and shape of the transmitted and received electric signals. Both of them are measurable and we can process them to extract pertinent information about the radar environment.

For the sake of simplicity, we will assume that the radar transmitter and receiver are located at the same point.

- Delay between emission and reception

We assume that the optical index of the atmosphere is 1 so that the radar wave velocity is c and that the distance between the radar antenna transmitter E and the target T is $R_{T}$. The total delay between the transmission and reception of the radar wave is:

(1) $$\Delta T=\Delta T_{1}+\Delta T_{2}=\frac{2R_{T}}{c}$$

Obviously, the distance to the target is fundamental during a measurement.

- Ambiguity

If the radar sends a periodical signal whose envelope has length duration $t_{p}$ and repetition period $T_{p}$, there exists a maximal distance ΔR called the unambiguous range for which a reflected pulse coming from one period cannot be confused with one of another period. It is given by:

(2) $$\Delta R<\frac{cT_{p}}{2}$$

The repetition period sets the maximal distance to the target during the evaluation of a radar.

- Radar resolution

The resolution D indicates the minimal distance the radar can measure:

(3) $$D=\frac{ct_{p}}{2}$$

Before evaluating a medical radar, we must be sure the bandwidth is sufficiently high to detect small movements such as heartbeats.

- Doppler Effect

We consider a target moving at speed v towards the radar from the initial position $R_{0}$, the radar being at the origin. The total delay ΔT between the transmission and reception of a radar wave is:

(4) $$\;\Delta T=\frac{2R_{0}}{c}{\left(1+\frac{v}{c}\right)}^{-1}$$

In practice, v≪c, so we can establish a Taylor development at the first order: “I confirm”

(5) $$\Delta T=\frac{2R_{0}}{c}\left(1-\frac{v}{c}\right)$$

Therefore, we must also know the target speed.

#### 2.2.2. Propagation in the Atmosphere

In reality, the radar transmission/reception phenomenon involves many energetic losses.

The signal-to-noise ratio SNR is given by:

(6) $$SNR=\frac{P_{t}G_{t}t_{p}\sigma \lambda^{2}G_{R}}{{\left(4\pi \right)}^{3}R^{4}LkT_{s}}$$

with transmitter power $P_{t}$, antenna directive gain $G_{t}$, length of a radar pulse $t_{p}$, target cross section σ, radar operative wavelength λ, antenna effective directivity $G_{R}$, distance to target R, losses factor L (circuit transmit loss, antenna loss, atmosphere loss, detection loss, etc.), Boltzmann constant k, and system noise temperature $T_{s}$.

The goal of our work is to evaluate the performance of an AI analysing the measurements of a radar. We do not want to evaluate the radar itself. Therefore, the interesting factors in the SNR are $G_{t},G_{R},\;\sigma ,R^{4},L,\mathrm{and}\;T_{s}$.

That is:

- $G_{t}$,$G_{R}$: angle between radar axis and target.
- σ: surface and reflectivity of the target.
- R: distance from target to radar.
- L: atmospheric and harware losses.
- $T_{s}$: operating temperature.

#### 2.2.3. Reflection or Propagation through Obstacles

Considering radar clutter, two situations arise: (1) if the radar is separated from the target by a wall, the radar will receive multiple phase-shifted waves due to multiple internal wall reflections, and (2) if there are walls around the target and the radar, there may also be multiple reflections. Therefore, we need to check if the radar deals well with these situations.

#### 2.2.4. Composition of Movements

We want to test the analogue medical radar on human targets. We can consider such targets as a set of non-deformable solids, limbs, attached via joint articulations to a deformable trunk on which we want to measure deformations linked to breathing and heart beating.

Each moving limb is a source of noise hiding the interesting movements. Therefore, considering, for instance, that the walking movement amplitude is $10^{-1}\;m$, which is large in comparison to breathing and heartbeat amplitudes ($\leq 10^{-2}\;m)$, it is important to test the medical radar in walking or other similar conditions to obtain a maximal target speed. This also means, as written previously, that the medical radar must have a sufficient resolution.

### 2.3. Comparison between Theory and Practice

We present in Table 2 the equivalences between the physical quantities of Section 2.1 and Section 2.2.

So far, we have not taken into account the following specificities of the targets:

- Nature of the target, human of machine.
- Age, height, weight of the target, if human.

Additionally, the following information concerning the realization of an experiment has not yet been taken into account:

- Duration of an experiment.
- Kind of breathing.
- Disturbances.
- Metrics to compare radar data with body sensors data.
- Reference sensors.

We will discuss some of these elements in the following sections.

## 3. Evaluation Equipment

In general, a radar measures the position and speed of moving objects in its field of view. Aiming at a human body, it is able to measure the trunk deformation.

The two deformations of interest here are the expansion/shrinkage of the lungs and chest during respiration and the smaller vibration prompted by a heartbeat. These two deformations of different magnitude ($10^{-2}$ m for respiration and $10^{-4}$ m for a heartbeat) provide useful medical information about the health of the target. Evaluating the performance of medical radars requires the development of a measurement platform to take reference measurements for comparison with the radar. 

We present here such a platform and perform the choice of reference signals. We perform our choice of sensors in Section 3.1, and that of the measurement platform in Section 3.2.

### 3.1. Choice of Sensors

We wanted to measure two variables: the heart and breathing rates. One of the main requirements for the measurement platform was the freedom to modify the software and ease of handling. Using devices recording their own data separately has the disadvantage of requiring physical handling to retrieve the data, and sometimes specific software to interpret these same data. Therefore, for a first prototype, we limited our choice of sensors to those that provide the rawest data.

Another requirement for the prototype was the low cost of the measurement platform and the speed of implementation. In order to meet this requirement, we resorted to “lean” prototyping, i.e., prototyping with few components. 

A final requirement was the need to compare the results of different sensors in order, if necessary, to select those that are robust to situations where there is movement. Regarding the resolution of the sensors, we do not need extremely high performance, since our goal is to measure the rates of two kinds of events, a heart beating and breathing, which can be considered as binary (pulse or not pulse), since we only want to obtain their rate. The resolution therefore only needs to be high enough to detect them. However, once we have the analogue intelligent radar, it will be interesting to check the spatial correlations between the radar and reference measurements.

#### 3.1.1. Sensors for the Breathing Rate

Breathing is a mechanism based on the lungs and the airways. The expansion/shrinkage of the lungs creates a pressure variation inside them, and the difference with the exterior atmosphere pressure generates an airflow. It also entails an expansion/shrinkage of the thoracic cage or the abdominal wall depending on the kind of breathing people practice.

Therefore, to obtain the breathing rate, we can either measure the airflow passing through the airways or the displacement of the thoracic cage/abdominal wall.

- Measuring the airflow

**Figure 1:**
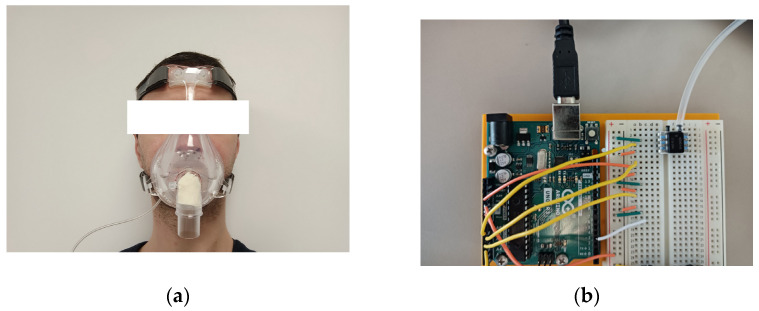
(**a**) A target wearing the mask with the pressure sensor tube coming from below; (**b**) pressure sensor on the right of the board with an Arduino Uno R3 board on the left. Equipment: For the prototyping, we used a Honeywell differential pressure sensor HSCDRRN005NDAA5 of ±5 in H_2_O ≈±12 mbar (for more accuracy, it is possible to reduce to ≈±5 mbar), a facial mask SEFAM Breeze and a silicone tube.

The medical community often resorts to spirometers to measure the breathing airflow. This tool also measures the respiration strength. We can refer to one disadvantage in addition to the requirement for software ownership, which is the handling of such a large system. A simpler solution is to use a simple differential pressure sensor. Such a sensor measures the pressure differences between two ports. We can then link one port to a volume connected to the human airways, and the other directly to the external atmosphere. In practice, it appeared easier to use a tube and insert it into the internal volume of a mask (Figure 1).

- Measuring the thoracic cage displacement

Video approaches are too expensive and inaccurate due to distances and angles, so it appears generally easier to measure the elongation of a material directly attached to the chest. Thus, breathing belts are often used. Their operating principle may be based on piezoelectric components or conductive yarn, the resistance of which varies with elongation.

**Figure 2:**
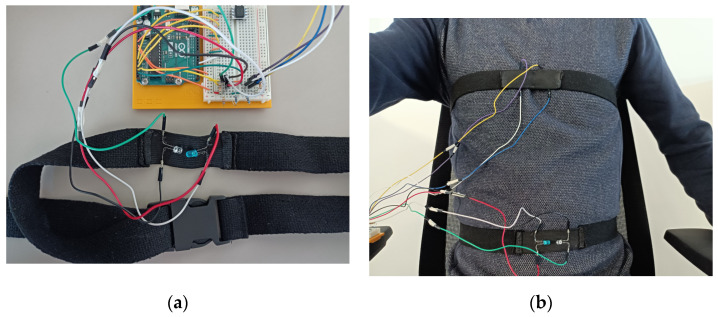
(**a**) Breathing belt with the phototransistor on the left of the stretchable strip and the LED on the right; (**b**) a target wearing two breathing belts, one on the chest, one on the belly. Equipment: 2 LEDs, 2 phototransistors, 2 elastic strips, 2 fabric belts with plastic buckles.

The problems with such tools are, first, the existence of a non-injective mathematical relationship between the resistance and the elongation, and second, the mechanical wear. This is why we resorted to an optical solution with one LED and a phototransistor. The phototransistor voltage is directly linked to the light intensity it receives from the LED and their linked resistor. After attaching these components to a belt, it becomes possible to measure the thoracic cage elongation. To achieve this, we sewed a non-stretchable belt on a stretchable strip. After this, we sewed the LED and the transistor onto the strip. To decrease the sensibility to the exterior light, we sewed another strip on the first one. We show below in Figure 2 this system with and without the second strip.

We report one problem regarding the upper belt experienced by women with breasts and a bra. Depending on the size and position of the breasts, we suggest positioning the upper belt either above or below them. For men, the upper belt can be put at nipple level. The lower belt was set at a mid-distance between the xiphoidal appendage and the belly button.

- Breathing measurements

**Figure 3:**
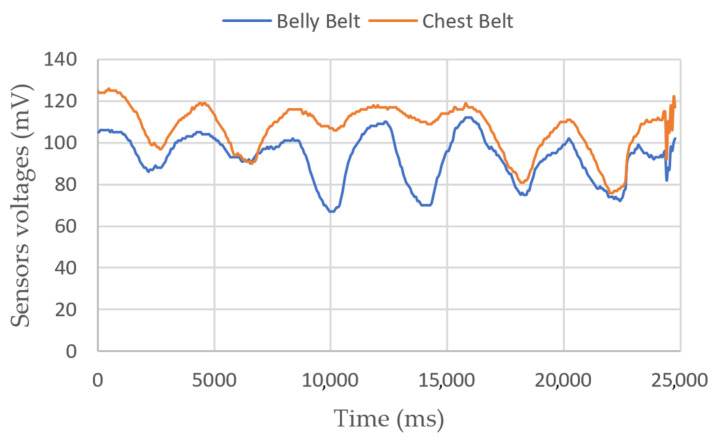
Belts sensors’ voltages vs. time. The figure shows 6 breathing cycles. The first two and last two breaths are performed with the belly and chest, and the middle two only with the belly.

**Figure 4:**
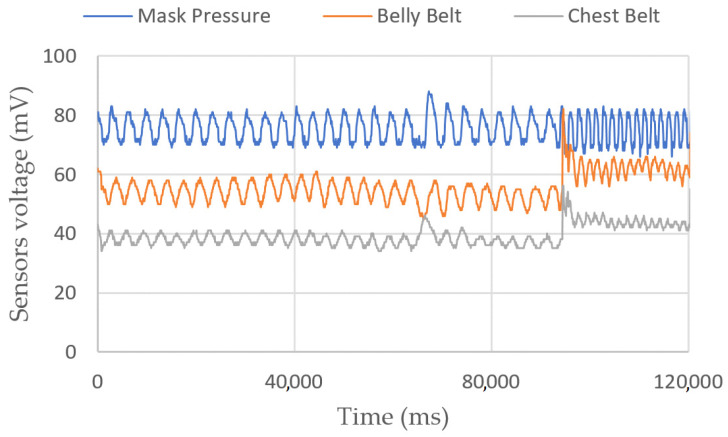
Belts and pressure sensors’ voltages vs. time. The target wanted to test various breathing styles, as shown by the strong inhalation near 70 s and the fast breathing after 90 s.

Breathing measurements on a sitting person are shown in Figure 3 and Figure 4. In the first figure, we show the belts’ elongation during six breathing cycles. The first and last two breaths were performed normally, and the middle two with the belly only. Depending on the person, the ratio between the amplitudes of variations can be different. In the second figure, we perform a 2-min test, with one strong inhalation and fast breathing at the end. We can see a mean altitude elevation in both belt sensors, showing a sudden increase in their voltages. We have not yet identified the origin of this phenomenon.

#### 3.1.2. Sensors for the Heart Rate

The heart beats to pump oxygenated blood everywhere in the organism. Such a beat is triggered by an electrochemical process during which the body endures small voltage differentials. We can measure such voltages with an electrocardiogram. Another solution is to measure the blood pulse directly on optically accessible blood veins. In this case, we can use green LED systems.

- Electrocardiograph

**Figure 5:**
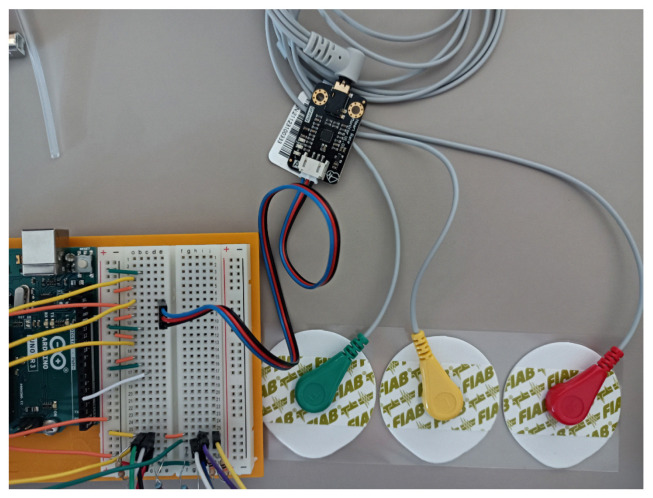
Arduino board with the heartbeat module in blue and 3 electrodes on the right. Equipment: DF-Robot Heart Rate Monitor Sensor SKU SEN0213 (DFRobot, Pudong, Shanghai, China).

We can measure the differential voltage on the body with electrodes directly stuck on the skin. The order of magnitude of this differential is $10^{-4}$ V. This relatively small voltage needs some amplification and filtering to be readable by a computer. Instead of making the circuit ourselves, we chose a circuit sold on the Internet. We integrated this circuit directly with an Arduino board, which is the computer we chose to use. On the other side, we connected wires and the electrodes. To detect heartbeats, Figure 5 shows the heartbeat module with the electrodes.

- Optical pulse sensors

**Figure 6:**
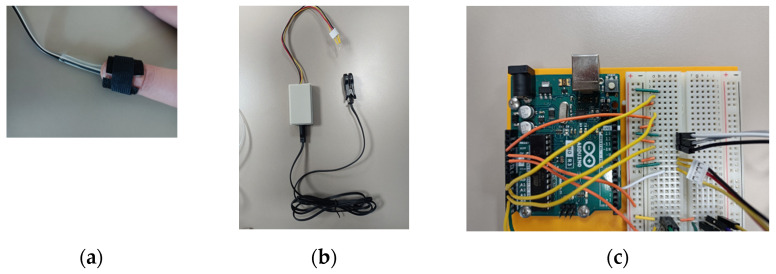
(**a**) The target’s finger with the finger heartbeat sensor tied with Velcro; (**b**) the ear clip heartbeat sensor; (**c**) the Arduino board with the electrical connections. Equipment: Joy-it SEN-KY039HS (Simac Electronics GmbH, D-47506 Neukirchen-Vluyn, Germany) and Seeed Grove-Ear-clip Heart Rate Sensor (Seeed Technology Co., Ltd., Nanshan, Shenzhen, China).

We chose to use two measurement points, one on a finger, and one on an ear. Sensors and wiring are shown in Figure 6.

We note that it is sometimes difficult to place the finger correctly on the pulse sensor. We also needed to make our own Velcro strip to tie the sensor to the finger. We recommend buying a sensor with a clip for better comfort. The measurement on ears has the small disadvantage of not being raw: it processes the signal and emits a yes-no answer on the presence of a pulse.

- Heart rate measurements

**Figure 7:**
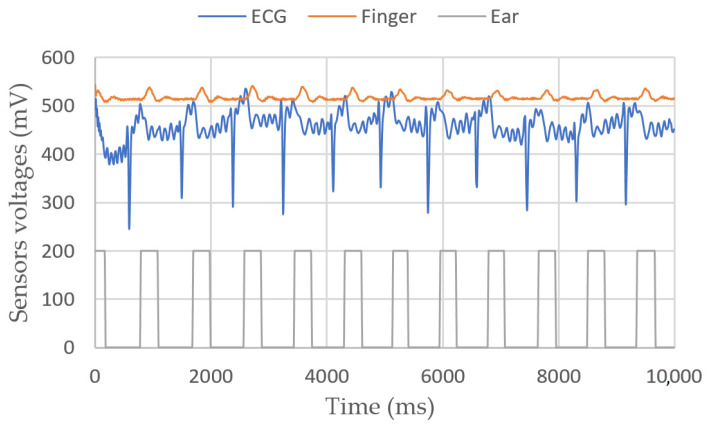
ECG, finger and ear heartbeat sensors’ voltages vs. time for a sitting person.

**Figure 8:**
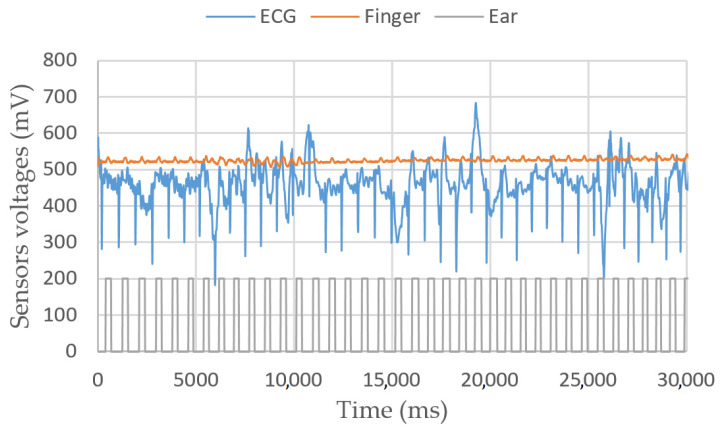
ECG, finger and ear heartbeat sensors’ voltages vs. time. In this second measurement, the target moves their finger between 5 and 10 s, their head between 15 and 20 s, their chest between 25 and 30 s.

**Figure 9:**
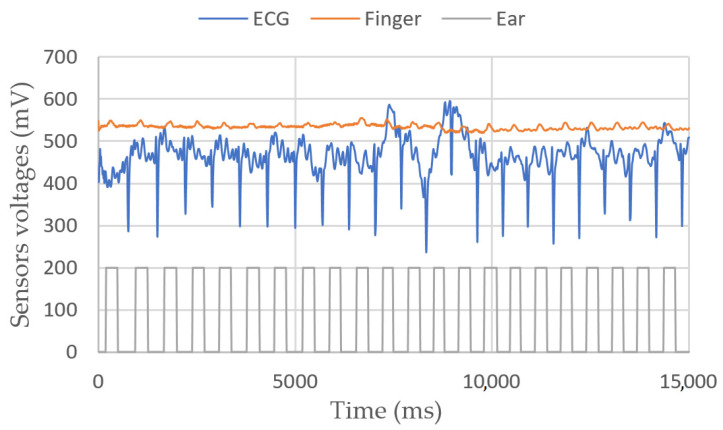
ECG, finger and ear heartbeat sensors’ voltages vs. time. In this third measurement, the target is standing and bending their knees multiple times between 5 and 10s.

Figure 7 shows a 10 s experiment on a sitting person. In Figure 8, the target moves its fingers and head to check that the sensors are working properly. In Figure 9, we simulate a walking person by asking them to bend their knees multiple times. Apparently, the peaks are still detectable.

##### 3.1.3. Circuit Diagrams

**Figure 10:**
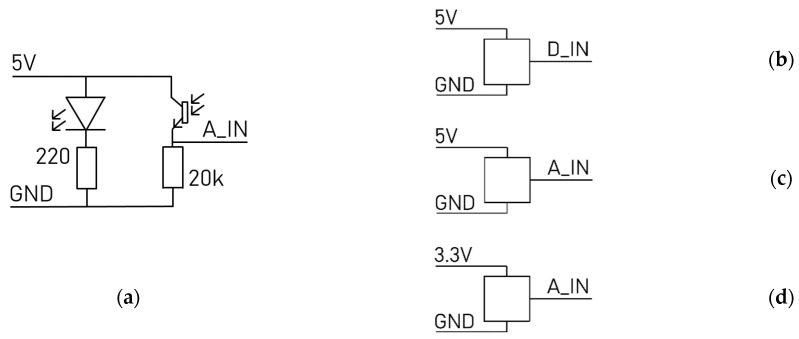
(**a**) Breathing belt circuit; (**b**) ear clip heartbeat sensor circuit; (**c**) pressure, finger heartbeat sensor circuit; (**d**) ECG circuit.

We show the overall structure for the circuits of the measurement platform in Figure 10.

### 3.2. Measurement Platform 

As already disclosed, we chose to use an Arduino board to gather the measurements. We chose it because of its low price and easiness of implementation. This resulted in a limitation in sample rate, resolution and current. 

On each measurement channel:

- The order of magnitude for sampling rate is 0.1 ms for an analogue input and 0.01 ms for a digital input, which is a sufficiently small time resolution to measure heartbeats (0.01 s) and breathing (1 s) [82];
- There are 10 bits for 5 V, that is, 5/1024 = 4.9 mV of resolution, which has been shown, after experiments, to be sufficiently small for a binary detection of heartbeats and breathing;
- The maximal current is 40 mA, which is sufficiently high for the working intensities of our sensors (ECG = 10 mA, ear clip = 7 mA, finger and LEDs = 20 mA, pressure = 5 mA, SD = 100 mA).

After this study, and some experiments, we considered that these limitations were not a problem for reaching our goal. 

#### 3.2.1. Choice of the ARDUINO Board

Two boards drew our attention—the basic Arduino R3 and the Arduino Uno WIFI REV2. The first one is sold with a beginner kit, which offers some electrical components and test cases to practice and train. However, the data are saved through a physical USB wire connection to a computer. This is not an issue if the human target is immobile, but it becomes problematic if the target walks. We would need, in this case, to extend the cable, which might interfere with the radar measurements because it might oscillate with the human movement at frequencies near the rates we want to measure. This is why we also selected the Arduino WiFi (Arduino s.r.l, 20900 Monza, Italy).

#### 3.2.2. Energy Supply

For motionless human targets, the basic Arduino R3 (Arduino s.r.l, 20900 Monza, Italy) can be used with a USB cable connected to a PC as a source of power. For moving people, we must attach a wearable energy supply onto the target if we do not want them to drag a wire. Batteries satisfy this condition. We plan to use the measurement platform during time intervals of varied lengths, and want the battery to be rechargeable. Therefore, we pre-selected two alternatives: a 9 V rectangle battery and LIPO battery. The first one is small with lower charge and is adapted for experiments shorter than one hour. The second can supply more current for an 8 h/day-long experiment. 

We also need to consider the battery health. In general, we reduce the aging of the battery by preventing it going under 20% charge, a number that can be generally translated in voltage terms as not going under 90% of the maximal voltage. Therefore, we need to measure this voltage and ensure that the Arduino checks the value and warns the user in case of discharge.

**Figure 11:**
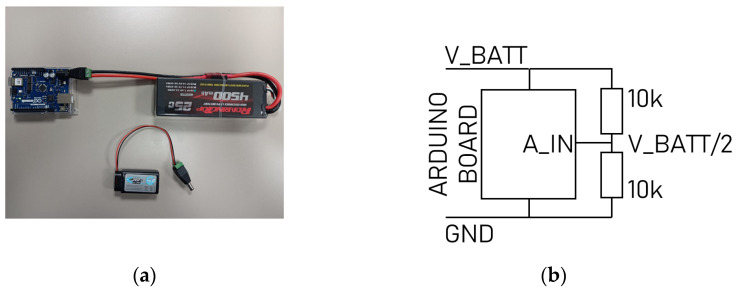
(**a**) Arduino WiFi R2 board in blue connected to a Lipo battery. Above, the NiMH battery; (**b**) electrical circuit for monitoring the battery voltage. Equipment: NiMH battery charger, 9 V NiMH battery 300 mAh, LiPo charger, LiPo 2S 4500 mAh battery.

For 9 V NiMH batteries, we consider the minimum acceptable voltage to be 7.9 V, and 7.4 V for LiPo 2S. We need to monitor the battery voltage with the Arduino. However, the measured voltages must be inferior to the Arduino supply voltage. To satisfy this condition, we can use a voltage divider with gain 0.5, for instance, which needs 2 identical resistors. The Arduino will compare the actual half battery voltage with 3.95 V and 3.7 V, respectively, and send a message by lighting a LED if the voltage goes below this level. See Figure 11 for batteries’ connections and the monitoring circuit.

#### 3.2.3. Data-Saving Mechanism

There are two ways to save data: either to have a data storage device on-board or sending it directly to the computer via WiFi.

- Using a SD card

**Figure 12:**
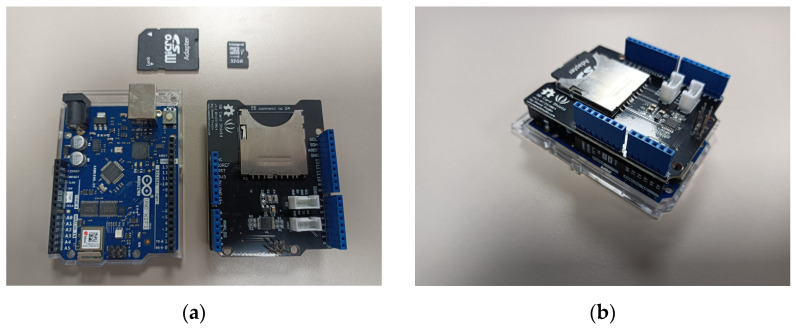
(**a**) Arduino WiFi R2 board on the left, SD card shield on the right, micro SD-card and its adaptor; (**b**) all elements from (**a**) assembled. Equipment: SD card shield from Seeed Studio, micro SD-card 32Go.

It is possible to buy on the Internet SD card adaptors directly connectable on Arduino boards. They are provided with computer code directly implementable on Arduino. The data can be saved in a .txt format file that is easy to use for signal processing (see Figure 12). The Arduino code can be found on the official Seeed Studio Wiki and also on the Arduino website [83,84].

- Using WIFI

**Figure 13:**
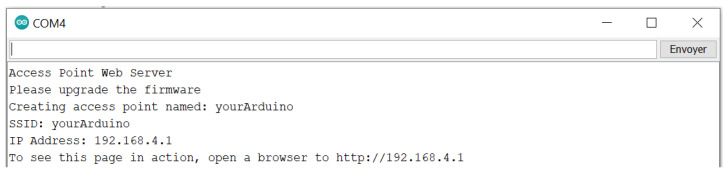
Arduino monitor showing the IP Address when creating the access point.

**Figure 14:**
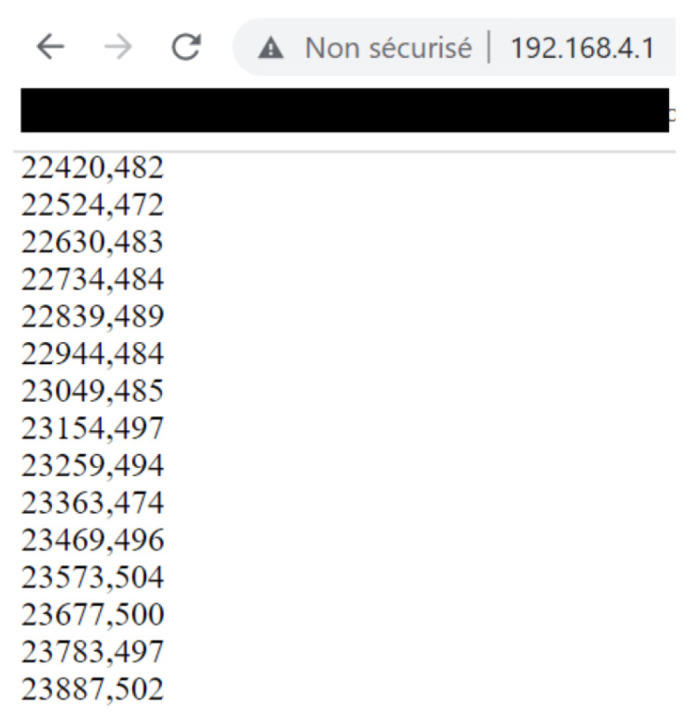
Browser window open on the IP address with printed data.

It is possible to set up a WiFi communication between a laptop and an Arduino WiFi. Here, we used the library WiFiNINA. The Arduino board creates an access point (Figure 13), and if a computer connects to it, it can receive data under the form of html code. We can access these data with any web browser and the Arduino network SSID and password (Figure 14). the access point creation sometimes failed, however, which we cannot yet explain.

The basic code can be found in web tutorials on the Arduino website [85,86].

- Storing the experimental data

The Arduino writes all the data in a .txt document. In the case the Arduino R3 is used, we need to open the Arduino software console at the beginning of the experiment and to unplug the USB cable at the end of it. It will be possible then to copy-paste the plotted data into the txt document.

In the case we use the Arduino WiFi, we plot the data on the html page accessible with a web browser, and copy and paste it into the .txt document.

## 4. Evaluation Protocol

The evaluation protocol describes the accurate conditions in which measurements with a medical radar are performed. We detail in Section 4.1. how we can design a scene with some of the physical quantities that we identified in Section 2.3. We provide examples in Section 4.1.1. We describe other experimental conditions in Section 4.1.2. Evaluation metrics are given in Section 4.2.

### 4.1. Installation of the Scene

**Figure 15:**
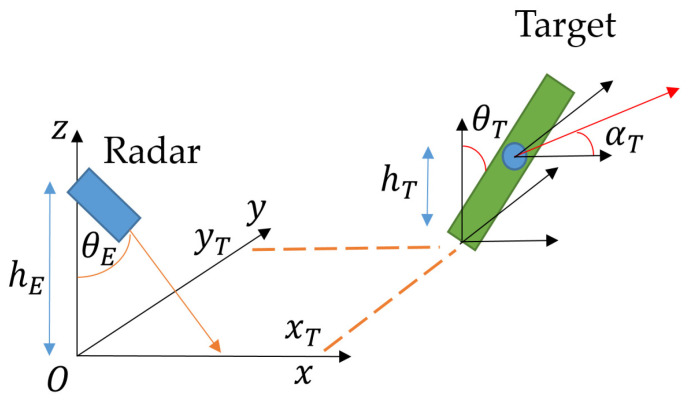
A scene with its descriptive variables.

The scene is the spatial layout of the various elements intervening in a measurement. In order to position the various objects of a scene, we introduce a mathematical referential, as shown in Figure 15.

The plane (Oxy) represents the ground of the laboratory. The (Oz) axis represents the vertical altitude line. The plane (Oxz) contains the central axis of the radar emission. $h_{r}$ represents the altitude from the ground of the radar, and $\theta_{E}$ represents the radar angle from the vertical line (Oz). $\theta_{E}=0$ means the radar is pointing at the ground. $\left(x_{c},y_{c}\right)$ is the value situating the foot of the target (Oxy). $h_{T}$ is the altitude of the target’s heart (or the periodic object). $\theta_{T}$ is the angle of the (foot–heart) axis to the vertical line. $\alpha_{T}$ is the body angle in (Oxy). For $\theta_{T}=0$ and $\alpha_{T}=0$, the target is standing up and its back is facing the plane (Oyz).

We then define a scene by a n-uplet of variables. In the following, these variables are stored in a first tensor defining the scene:

(7) $$S_{RT}=\left(h_{E}\;;\;\theta_{E}\;;\;X_{T}\;;\;Y_{T}\;;\;H_{T}\;;\;T_{T}\;;\;A_{T}\right)$$

This tensor contains three new column vectors: $X_{T}\;;\;Y_{T}\;;\;H_{T}\;;\;T_{T}\;;\;A_{T}$. These vectors contain, respectively, the various respective positions $x_{Ti}\;;\;y_{Ti}\;;\;h_{Ti}\;;\;\theta_{Ti}\;;\;\alpha_{Ti}$ of multiple possible targets. Simply defined by $S_{RT}$, the scene contains only a radar and multiple targets. 

To specify the presence of obstacles, we introduce a new tensor $S_{o}$ where the different values $x_{oi}\;;\;y_{oi}\;;\;b_{oi}\;;\;h_{oi}$ describing the position of the obstacles are referenced by the index i and arranged in column vectors $X_{o}\;;\;Y_{o}\;;\;B_{0}\;;\;H_{o}$:

(8) $$S_{o}=\left(X_{o}\;;\;Y_{o}\;;\;B_{0}\;;\;H_{o}\right)$$

**Figure 16:**
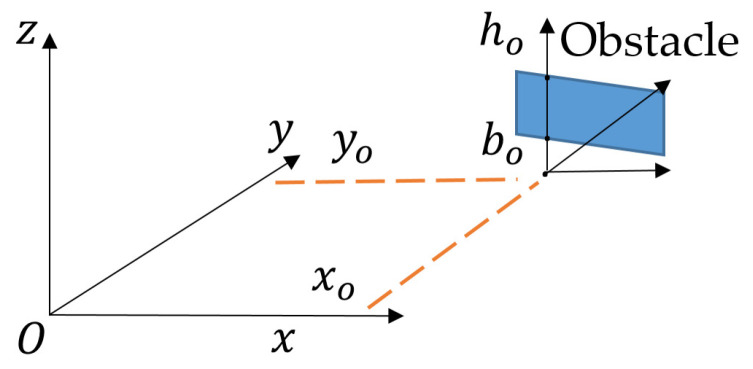
A scene with its obstacle.

Figure 16 shows these various values expressed in the same referential (Oxyz) as before.

An obstacle is defined by a set of segments for which the projection on (Oxy) is located on $x_{o}\;;\;y_{o}$ and height is between $b_{o}$ and $h_{o}$. These segments are linked via a parametric equation setting the values of the last four parameters. The obstacle width is given in the (xy) plane according to the considered scenario, and is centred on the ($b_{0}h_{0})$ axis. This representation enables us to describe walls, floors or ceilings, and also ground rubble.

In the case where a scene object is moving, the values inside the tensors $S_{rc}$ and $S_{o}$ receive the parenthesis (t) to signify the dependence on time of the corresponding variable.

#### 4.1.1. Detail of the Scenarios

The scenarios represent moving or static scenes during which a radar signal is measured.

If multiple values between a and b spaced by an increment c of a scene parameter are to be tested, we will write them between braces {a :c :b}. The value of a parameter can also be denoted in an interval [a,b]. This will be written with a ∈ symbol. In the case we want to describe the arc defining a long object, the symbol = will be used. The meter and the degree are taken as spatial unities. If not specified, the atmosphere is that of the laboratory with temperature T∈[283,303]K.

In what follows, we present a few typical scenarios as examples. We plan to test more extreme scenarios in order to evaluate the radar limits.

Scenario 1: standing target, various distances (to check performances with distance)

$\begin{array}{c} \;\;\;\;\;\;\;S_{RT}=(h_{E}=1\;;\;\theta_{E}=90\;;\;X_{T}=\left\{1:2:11\right\}\;;\;Y_{T}=0;\;H_{T}\;\in \left[1,2\right]\;;\;T_{T}=0\;;\;\\ \;\;\;\;\;\;\;\;\;\;\;\;\;\;A_{T}=0) \end{array}$

Scenario 2: lying-down target, various angles (to check body orientation effects)

$\begin{array}{c} \;\;\;\;\;\;\;S_{RT}=(h_{E}=1\;;\;\theta_{E}=90\;;\;X_{T}=\left\{5\right\}\;;\;Y_{T}=0;\;H_{T}\;\in \left[0,1\right];\;T_{T}=90\;;\;\\ \;\;\;\;\;\;\;\;\;\;\;\;\;\;A_{T}=\left\{0:45:180\right\}) \end{array}$

Scenario 3: standing target, after running (to watch heartbeat and respiratory rates variations)

$\;\;\;\;\;\;\;S_{RT}=\left(h_{E}=1\;;\;\theta_{E}=90\;;\;X_{T}=5\;;\;Y_{T}=0;\;H_{T}\;\in \left[1,2\right]\;;\;T_{T}=0\;;\;A_{T}=0\right)$

Scenario 4: walking target (accuracy for walking target)

$\begin{array}{c} \;\;\;\;\;\;\;S_{RT}=(h_{E}=1\;;\;\theta_{E}=90\;;\;X_{T}\left(t\right)=4.\left(1+\frac{3}{4}.\cos\left(0.2t\right)\right)\;;\;\\ \;\;\;\;\;\;\;\;\;\;\;\;\;\;Y_{T}\left(t\right)=4.\left(1+\frac{3}{4}.\sin\left(0.2t\right)\right)\;;\;H_{T}\;\in \left[1,2\right];\;T_{T}=0\;;\;A_{T}\left(t\right)=90+0,3.t) \end{array}$

Scenario 5: moving radar (moving radar effect. In case someone handles the radar)

$\begin{array}{c} \;\;\;\;\;\;\;S_{RT}=(h_{E}=1+0,5.\cos\left(0,3.t\right)\;;\;\theta_{E}=90\;;\;X_{T}=5\;;\;Y_{T}=0\;;\;H_{T}\in \left[1,2\right];\;\\ \;\;\;\;\;\;\;\;\;\;\;\;\;\;T_{T}=0\;;\;A_{T}=0) \end{array}$

Scenario 6: multiple static targets. Various distances, no alignments (for multiple frequencies extraction)

$\;\;\;\;\;\;\;S_{RT}=(h_{E}=1\;;\;\theta_{E}=90\;;\;X_{T}=\left(\begin{array}{c} 7\\ 3\\ 5 \end{array}\right)\;;\;Y_{T}=\left(\begin{array}{c} 2\\ 0\\ 3 \end{array}\right)\;;\;H_{T}\;\in \left(\begin{array}{c} \left[1,2\right]\\ \left[1,2\right]\\ \left[1,2\right] \end{array}\right);$

$\;\;\;\;\;\;\;\;\;\;\;\;\;\;T_{T}=\left(\begin{array}{c} 0\\ 0\\ 0 \end{array}\right)\;;\;A_{T}=\left(\begin{array}{c} -90\\ 0\\ 135 \end{array}\right))$

Scenario 7: static target, three close targets (to test the resolution)

$\;\;\;\;\;\;\;S_{RT}=(h_{E}=1\;;\;\theta_{E}=90\;;\;X_{T}=\left(\begin{array}{c} 4\\ 4,2\\ 4,4 \end{array}\right)\;;\;Y_{T}=\left(\begin{array}{c} 0,2\\ 0\\ -0,2 \end{array}\right)\;;\;H_{T}\in \left(\begin{array}{c} \left[1,2\right]\\ \left[1,2\right]\\ \left[1,2\right] \end{array}\right);\;$

$\;\;\;\;\;\;\;\;\;\;\;\;\;\;T_{T}=\left(\begin{array}{c} 0\\ 0\\ 0 \end{array}\right)\;;\;A_{T}=\left(\begin{array}{c} -180\\ -180\\ -180 \end{array}\right))$

Scenario 8: static target, 20cm-width wall (wall reflections and attenuation effects)

$\begin{array}{c} \;\;\;\;\;\;\;S_{RT}=(h_{E}=1\;;\;\theta_{E}=90\;;\;X_{T}=\left\{4:1:8\right\}\;;\;Y_{T}=0;\;H_{T}\;\in \left[1,2\right]\;;\;T_{T}=0\;;\;\\ \;\;\;\;\;\;\;\;\;\;\;\;\;\;A_{T}=0)\\ \;\;\;\;\;\;\;S_{o}=\left(X_{o}=2\;;\;Y_{o}=\left[-1,1\right]\;;\;B_{0}=0\;;\;H_{o}=2\;\right) \end{array}$

Scenario 9: standing target, talking person (robustness against noise)

$\;\;\;\;\;\;\;S_{RT}=\left(h_{E}=1\;;\;\theta_{E}=90\;;\;X_{T}=\left\{3,7\right\}\;;\;Y_{T}=0;\;H_{T}\;\in \left[1,2\right]\;;\;T_{T}=0\;;\;A_{T}=0\right)$

Scenario 10: foggy atmosphere in climatic chamber (radar wave attenuation)

$\;\;\;\;\;\;\;S_{RT}=\left(h_{E}=1\;;\;\theta_{E}=90\;;\;X_{T}=5\;;\;Y_{T}=\left\{0\right\};\;H_{T}\;\in \left[1,2\right]\;;\;T_{T}=0\;;\;A_{T}=0\right)$

This mathematical representation may seem dry at first sight, but this is a small price to pay to define a scenario accurately.

##### 4.1.2. Experiment Duration and Breathing

Each scenario is evaluated for 120 s (thirty breaths or so). The targets should breathe normally for 60 s, slowly for 30 s, and quickly for 30 s. We have not yet thought about more complicated breathing that could put a medical radar in difficulty.

### 4.2. Evaluation Metrics

The evaluation equipment and protocol may be applied to any medical radar. It is in the metrics that the notion of analogue intelligent radar appears. 

#### 4.2.1. Measured Variables

We compare the reference signals with the radar signal in all scenarios. We also measure the AI chip intensity voltage for the calculation of the energy consumption. The various measurements to be performed are:

- the sample time $t_{k};$
- the heart rate sensors signals $h_{ref;}$
- the breathing rate sensors signals $b_{ref};$
- the AI value for heartbeat frequency $f_{h,AI};$
- the AI value for breathing, scalar mean $f_{b,AI}\left(\tau \right);$
- the AI supply current $I_{r};$
- the AI supply voltage $V_{r}.$

#### 4.2.2. Extraction and Comparison of Body Frequencies

As explained in Section 2, we focus in this article on the good match between information measured on the human body and the AI result. The protocol of Section 4 and the sensors of Section 3 allows the data recovery in a txt format. From this point, we need a program to extract the heart and breathing rates from measurements $h_{ref}$ and $b_{ref}$. To achieve this, we can follow either a frequency approach (detection of frequency peaks) or a temporal approach (detection of high amplitude gradient). We have not written and tested such a program yet.

Let $f_{b,AI}\left(\tau_{k}\right)$ and $f_{h,AI}\left(\tau_{k}\right)$ be the frequencies given by the radar AI for breathing and heart rate, and $f_{b,ref}\left(\tau_{k}\right)$ and $f_{h,ref}\left(\tau_{k}\right)$ be the frequencies calculated from measurement. These signals depend on time $\tau_{k}$, which is a discrete time associated with the beginning of every new time period on which a frequency is derived (so it differs from $t_{k}$). Their comparison could be made by evaluating the squared difference of corresponding frequencies:

(9) $$\varepsilon_{b}=\underset{\tau_{k}}{\sum }{\left(f_{b,AI}\left(\tau_{k}\right)-f_{b,ref}\left(\tau_{k}\right)\right)}^{2}$$

(10) $$\varepsilon_{h}=\underset{\tau_{k}}{\sum }{\left(f_{h,AI}\left(\tau_{k}\right)-f_{h,ref}\left(\tau_{k}\right)\right)}^{2}$$

We do not know yet on which time window the AI computation will operate, so we cannot be more accurate. We will also need to compare the error rates of intelligent radars with that of normal radars in order to assess the benefit of using AI.

#### 4.2.3. Energy Consumption

Concerning the energy consumption, one needs to multiply the instantaneous values of $V_{r}$ and $I_{r}$ and integrate this with time. The formula is:

(11) $$E=\int_{0}^{120s}V_{r}\left(t\right)I_{r}\left(t\right)dt=\overset{n_{smp}}{\underset{k=0}{\sum }}\left(t_{k+1}-t_{k}\right)V_{r}\left(k\right)I_{r}\left(k\right)$$

where, in the second equality, $n_{smp}$ is the number of samples measured during the 120 s experiment. The time delay between each sample may not be the same. 

We cannot give more details on the way to measure these voltages and currents now because the analogue intelligent radar is still in the prototyping phase. Ideally, this should be performed using the same measurements.

#### 4.2.4. Analogue Circuit Processing Time

For the same reason, it is difficult to assert accurately now how we are going to measure the analogue circuit processing time, since it will continuously receive information from the radar antennas. At first sight, we can imagine the generation of a periodical voltage signal simulating the antennas signals and measure with an oscilloscope the phase shift between the input of the analogue AI board and its output, which should also be periodical.

In addition to this, we plan to benchmark multiple microcomputers to compare their energy consumption and their processing time (we can get the latter from their internal clocks) with those of the analogue radar.

## 5. Discussion

Overall, we have presented the principles of development of a systematic evaluation protocol for medical radars on a theoretical and experimental basis. We have also presented experimental equipment and metrics to perform the evaluation. We still lack an actual evaluation with an analogue intelligent radar to refine these elements. This radar is currently at the design stage, and so we do not yet have results to compare our protocol with those found in the literature.

Meanwhile, we still have some topics to study:

- We need to develop the tools to analyse and compare the radar and reference results.
- We have to wait for the analogue intelligent medical radar prototype to finalise our metrics for energy consumption and processing delay and to evaluate the effectiveness of our protocol.
- We plan to benchmark the prototype with multiple different digital platforms. Coming back to Section 2 and the distinction between the three levels of information, we stated that in this paper we were studying the matching between levels (1) and (3) (body information and AI results). It is also possible to check the quality of the matching between levels (2) and (3) and, in a way, the measurements of the processing delay and the energy consumption already belong to this relationship. The benchmarking also belongs to it, since it will be made for all computers based on the same data and frequency extraction algorithm.
- Concerning the evaluation protocol, we have not worked yet on a puppet to simulate a respiration and heartbeats. Such a puppet could potentially perform more specific and regular movements than human can do.
- We also have to check how the Arduino WiFi interacts with the radar.
- We did not talk either of the effect of radar jamming. It could be interesting to see how the AI works with this kind of electromagnetic disturbance, as this may occur in some situations, for example in military applications. The same remark applies to the kind of clothing the target must wear during the experiment. We need additional experiments to decide how these two elements should be included in our protocol.

## Figures and Tables

**Table 2:** Equivalences between physical quantities.

Section 2.1.	Section 2.2.
Number of targets	Only one in the presented section
Distance and position of the target	Distance *R*
Movement of the target	Speed v
Alignment of the radar	$G_{t}$, $G_{R}$
Position of the radar	0 in previous examples
Target clothes	Surface and reflectivity of the target σ
Atmosphere quality	Atmosphere loss L and operating temparature $T_{s}$
Room	Reflections on walls
Obstacle	Width

## Data Availability

Not applicable.
